# Sensation Seeking and Online Gaming Addiction in Adolescents: A Moderated Mediation Model of Positive Affective Associations and Impulsivity

**DOI:** 10.3389/fpsyg.2017.00699

**Published:** 2017-05-05

**Authors:** Jianping Hu, Shuangju Zhen, Chengfu Yu, Qiuyan Zhang, Wei Zhang

**Affiliations:** ^1^Laboratory for Behavioral and Regional Finance, Guangdong University of FinanceGuangzhou, China; ^2^School of Psychology and Center for Studies of Psychological Application, South China Normal UniversityGuangzhou, China; ^3^School of Education and Center for Mind and Brain Science, Guangzhou UniversityGuangzhou, China; ^4^Guangzhou College of CommerceGuangzhou, China

**Keywords:** sensation seeking, positive affective associations, impulsivity, online gaming addiction, adolescence

## Abstract

Based on the Dual Systems Model (Somerville et al., 2010; Steinberg, 2010a) and the biosocial-affect model (Romer and Hennessy, 2007) of adolescent sensation seeking and problem behaviors, the present study examined how (affective associations with online games as a mediator) and when (impulsivity as a moderator) did sensation seeking influence online gaming addiction in adolescence. A total of 375 Chinese male adolescents (mean age = 16.02 years, *SD* = 0.85) from southern China completed anonymous questionnaires regarding sensation seeking, positive affective associations with online games, impulsivity, and online gaming addiction. Our findings revealed that sensation seeking, positive affective associations with online games and impulsivity were each significantly and positively associated with online gaming addiction in adolescents. Positive affective associations mediated the relationship between sensation seeking and online gaming addiction. Further, impulsivity moderated the relationship between positive affective associations and online gaming addiction, such that the association between positive affective association and online gaming addiction was stronger for high than for low impulsivity adolescents. These findings underscore the importance of integrating the biosocial-affect model and the Dual Systems Model to understand how and when sensation seeking impacts adolescent online gaming addiction.

## Introduction

With ever more people having convenient access to high-speed internet, online gaming has become increasingly popular, particularly among adolescents. As the popularity of online games has grown, so have concerns over the outcomes of excessive usage. Just like addiction to alcohol or drugs, addictive gamers show several classic signs of addiction, including being preoccupied by computer games, withdrawing from social life to play games, using games to escape from the pressure in the real world (Kuss and Griffiths, 2012; Mai et al., 2012; Gunuc, 2015). Online gaming addiction has become a serious public health concern around the world, especially in China and other Asian countries (Block, 2008). It is urgent to understand the psychological mechanisms of online gaming addiction, which are the basis of prevention and intervention.

Internet addiction is associated with an increased prevalence of externalizing problem behaviors (e.g., substance use and sexual intercourse; Ko et al., 2008; Sung et al., 2013), and internalizing problem behaviors (e.g., depression and social anxiety; Ko et al., 2014). These problem behaviors have been shown to be significantly related to sensation seeking (Smith et al., 1992; Crawford et al., 2003; Mann et al., 2015; Engel-Yeger et al., 2016). Sensation seeking describes the willingness and actions of taking risks to attain novel and highly stimulating experiences (Zuckerman, 1979; Steinberg et al., 2008). It is an intriguing personality trait, which can serve as a risk and protective factor for certain problem behaviors (Norbury and Husain, 2015). Although there is accumulated evidence on the positive effect of sensation seeking on adolescents’ internet addiction (Ko et al., 2006; Li et al., 2010, 2016; Bitton and Medina, 2015), few studies have examined the relationship between sensation seeking and online gaming addiction (Mehroof and Griffiths, 2010). Moreover, it remains largely unclear how (i.e., the mediating mechanism) and when (i.e., the moderating mechanism) does sensation seeking influence online gaming addiction. Addressing these issues are key not only to understanding the etiology of online gaming addiction but also to developing effective intervention programs (Li et al., 2010).

### Affective Associations as a Mediator

Biosocial-affect model of adolescent problem behavior (Romer and Hennessy, 2007) proposed that, adolescents’ sensation seeking may influence their affective associations with the behavior, which may further influence their risk taking. Following Kiviniemi et al. (2007), affective associations refer to the feelings associated with a specific stimulus or behavior. Consistent with this theoretical framework, a few studies have demonstrated that positive associations with risky behavior emerged as a significant mediator of sensation seeking on drug use (Romer and Hennessy, 2007) and alcohol use (Urbán et al., 2008). However, there is a need for empirical research to investigate whether this model can be applied to online gaming addiction in adolescence.

Some indirect evidence has implied that positive affective associations mediate the relationship between sensation seeking and online gaming addiction in adolescence. Positive reactions (including cognitive and socio-affective) to novelty and stimulating experiences have been an essential part of definition of sensation seeking (Zuckerman, 1996). Recent evidence has also shown that players with high sensation seeking found computer games more entertaining than players with low sensation seeking (Fang and Zhao, 2010). On the other hand, recent studies have shown that positive affective associations may impact one’s addictive behavior (Karlsson, 2012; Stautz and Cooper, 2015). For example, Lee et al. (2014) found that perceived enjoyment and the associated positive affect positively influenced the development of excessive use of online games. Tone et al. (2014) reported that online game attraction was positively related to online gaming addiction. Taken together, sensation seeking may be linked to positive associations with online games, which in turn is linked to online gaming addiction. However, to date, no known studies have yet directly examined the mediating role of positive affective associations with online games in the link between sensation seeking and online gaming addiction in adolescence.

### Impulsivity as a Moderator

Based on human neuroimaging findings, the Dual Systems Model (Somerville et al., 2010; Steinberg, 2010a) was developed to explain why adolescents engage in problematic behaviors. Increasing problematic behaviors in adolescence are the product of the more mature limbic (socioemotional) system winning over the prefrontal (cognitive control) system (Steinberg et al., 2008; Steinberg, 2010a). More and more research has begun to link the changes in neural systems to changes in behavior in adolescence (Steinberg et al., 2008; Steinberg, 2010b; Harden and Tucker-Drob, 2011; Quinn and Harden, 2013; Willoughby et al., 2013). For example, cross-sectional (Steinberg et al., 2008) and longitudinal (Harden and Tucker-Drob, 2011) research has shown that age differences in sensation seeking and impulsivity match age differences in socioemotional and cognitive control neural systems, respectively. Quinn and Harden (2013) reported that, age-related changes in sensation seeking and impulsivity were associated with changes in substance use. These findings provide behavioral evidence for the Dual Systems Model. However, these studies did not directly test how these two systems interact to influence adolescents’ problematic behavior. Whereas sensation seeking is thought to stem from sensitivity of the socio-emotional system to affective cues, impulsivity is suggested to stem from the poor performance of the cognitive control system (Steinberg et al., 2008; Harden and Tucker-Drob, 2011; Quinn and Harden, 2013). The present study introduced the variable of impulsivity and investigated whether impulsivity moderates the relationship between socio-emotional system and problem behavior, directly testing how socioemotional and cognitive control systems interact to influence adolescents’ online gaming addiction.

Currently, little is known about the moderating role of impulsivity in the link between affective associations and online gaming addiction, despite considerable evidence suggesting that impulsivity moderates the relationship between affect and drinking variables (Colder and Chassin, 1997; Lindgren et al., 2014). For example, Colder and Chassin (1997) showed the role of impulsivity as a moderator of the link between positive affectivity and alcohol consumption. In a recent study, self-control (which shows conceptual overlap with impulsivity) was proposed as a moderator of the impact of implicit affective associations on alcohol use (Lindgren et al., 2014). Based on these aforementioned findings and the Dual Systems Model, it is reasonable to infer that impulsivity moderates the relationship between affective associations with online games and online gaming addiction.

In summary, based on the biosocial-affect model of adolescent problem behavior and the Dual Systems Model, the current study sought to reveal the underlying mechanisms of the relationship between sensation seeking and online gaming addiction with two specific goals: (1) to examine whether affective associations with online games mediate the relationship between sensation seeking and adolescents’ online gaming addiction, and (2) to test whether the relationship between affective associations and online gaming addiction is moderated by the individual trait of impulsivity. Therefore, two hypotheses could be proposed as follows:

Hypothesis 1: sensation seeking would increase positive affective associations with online games, which in turn contributes to online gaming addiction in adolescence.
Hypothesis 2: the individual trait of impulsivity would moderate the impact of positive affective associations on online gaming addiction, such that the relationship between positive affective associations and online gaming addiction would be stronger for adolescents with high as compared to low impulsivity.

In addition, there is lack of evidence on whether the relationship between sensation seeking and positive affective associations or the relationship between sensation seeking and online gaming addiction is moderated by impulsivity. Thus, we do not propose specific hypotheses regarding these relationships.

## Materials and Methods

### Sample

Male adolescents are particularly at-risk for online gaming addiction (Li and Wang, 2013; Ha and Hwang, 2014; Wang et al., 2015). To allow more precise inference for this group, this study was conducted only on male adolescents. The original sample consisted of 413 male adolescents from Grade 10 and 11 in southern China. Of these, 38 (9.2%) were excluded because they had no experience playing online games, resulting in the current sample of 375 male adolescents. The mean age of this sample was 16.02 years (SD, 0.85 years), ranging from 15 to 17 years.

### Measures

#### Sensation Seeking

Adolescents’ sensation seeking was assessed by a short form of sensation seeking scale, which demonstrated reliability and validity (Steinberg et al., 2008; Li et al., 2010). It consists of six items, which are scored on a six-point scale ranging from 1 (almost always untrue of you) to 6 (almost always true of you). Higher means represent higher levels of sensation seeking. Cronbach’s α for the present sample was 0.68.

#### Impulsivity

Adolescents’ impulsivity was assessed by three six-item subscales from the Barratt Impulsiveness Scale, Version 11 (Patton and Stanford, 1995) also used in Steinberg et al. (2008). Forward- and back-translation procedures were conducted to build the Chinese version of the measurement. Each item was scored on a four-point scale ranging from 1 (rarely/never) to 4 (almost always/always). Subscales were averaged to form a total impulsivity score. A higher mean represents a higher level of impulsivity. Cronbach’s α coefficient for the present sample was 0.65.

#### Affective Associations

In a pilot study, we used the affect pool measure developed by Peters and Slovic (1996) to measure the affect toward online games (or gaming). Fifty online gaming players were recruited (46 male; mean age ± SD, 17 ± 2.03). They were asked to report the first three words that came to mind when they were told to think of online gaming. They then rated each word on a five-point scale ranging from very negative to very positive. The top seven most frequent words listed by these players were happy, interesting, attractive, popular, relaxing, concentrating, and making friends. We then used these seven words to make sentences in the current study. For example, “when playing online games, I feel happy.” Participants rated how true each statement was for themselves on a six-point scale ranging from 1 (almost always untrue of you) to 6 (almost always true of you). These seven words were positive, thus, a higher mean represents more positive affective association with online gaming. Cronbach’s α coefficient for the present sample was 0.90.

#### Online Gaming Addiction

The online gaming addiction scale was modified from the Revised Chinese Internet Addiction Scale (CIAS) (Chen et al., 2003) to measure the degree of online gaming addiction tendency in participants. The scale has 26 items and consists of two subscales: Core Symptoms and Related Problems. The former includes three dimensions: compulsive use, withdrawal, and tolerance; the latter includes two dimensions: interpersonal and health-related, and time management problems. For each item, participants indicated how true each statement was for themselves on a four-point scale ranging from 1 (almost always untrue of you) to 4 (almost always true of you). The mean was taken with a higher mean representing a higher level of online gaming addiction. Cronbach’s α coefficient of the scale and the two subscales in the study was 0.94, 0.91, and 0.87, respectively.

### Procedures

Informed consents were obtained from the school, all participants and their parents. Participants in this study were voluntary and anonymous. They were given approximately 30 min to complete the questionnaires in their classrooms. All materials and procedures were approved by South China Normal University Human Investigation Committee.

### Statistical Analysis

First, we presented descriptive statistics and bivariate correlations for the major variables. Second, to test Hypothesis 1, we followed MacKinnon’s (2008) four-step procedure to evaluate the mediation effect. Third, to test Hypothesis 2, we followed Muller et al’s (2005) description regarding the evaluation of moderated mediation.

## Results

### Preliminary Analyses

Means, standard deviations, and the correlation matrix of the major variables are presented in **Table 1**. Sensation seeking, positive affective associations with online gaming and impulsivity showed significant and positive correlations with core symptoms and related problems of online gaming addiction, suggesting that all three of these factors are risk factors for online gaming addiction. Sensation seeking correlated positively with positive affective associations; however, the correlation of impulsivity and affective associations was not significant.

**Table 1:** Means and standard deviations of the major variables along with their correlations.

Variables	Range	*M*	*SD*	1	2	3	4	5
(1) Sensation seeking	1~6	3.19	0.98	—				
(2) Positive affective associations	1~6	3.64	1.07	0.199^∗∗^	—			
(3) Impulsivity	1~4	2.22	0.34	0.147^∗∗^	0.087	—		
(4) Core symptoms	1~4	1.92	0.58	0.152^∗∗^	0.463^∗∗^	0.312^∗∗^	—	
(5) Negative outcomes	1~4	1.95	0.57	0.105^∗^	0.386^∗∗^	0.272^∗∗^	0.823^∗∗^	—

*N = 375; *^∗^p* < 0.05, *^∗∗^p* < 0.01.*

In Hypothesis 1, to evaluate the mediating effect of affective associations with online games, MacKinnon’s (2008) four-step procedure was used. The first three steps were to test the direct effect using linear regression, including (1) a link between sensation seeking and online gaming addiction; (2) a link between sensation seeking and positive affective associations with online games; (3) a link between positive affective associations with online games and online gaming addiction while controlling for sensation seeking. All links in these three steps should prove significant. In the fourth step, sensation seeking and positive affective associations with online games were entered in the linear regression model. We used the Sobel test to determine whether the effect of positive affective associations with online games remains significant.

The results for the mediation model examining the relationship between sensation seeking, affective associations with online games, and online gaming addiction are presented in **Table 2**. The effect of sensation seeking on online gaming addiction (*b* = 0.152, *p* < 0.01), the effect of sensation seeking on affective associations with online games (*b* = 0.199, *p* < 0.001), and the effect of affective association on online gaming addiction (*b* = 0.463, *p* < 0.001) were significant. In the fourth step, when controlling for sensation seeking, the effect of affective associations on online gaming addiction was significant (*b* = 0.450, *p* < 0.001); however, the effect of sensation seeking on online gaming addiction was no longer significant (*b* = 0.062, *p* > 0.1). Finally, the Sobel test indicated that the full mediation effect of affective associations with online games on the relationship between sensation seeking and online gaming addiction was significant (*Z* = 3.63, *p* < 0.001). Hypothesis 1 was supported.

**Table 2:** Testing the mediation effect of sensation seeking on online gaming addiction in adolescence.

	Model 1 (criterion online gaming addiction)		Model 2 (criterion positive affective associations)		Model 3 (criterion online gaming addiction)	
Predictors	*b*	*t*	*b*	*t*	*b*	*t*
Sensation seeking	0.152	2.963^∗∗^	0.199	3.918^∗∗∗^	0.062	1.328
Positive affective associations					0.450	9.624^∗∗∗^
*R^2^*	0.023		0.040		0.218	
*F*	8.780^∗∗^		15.354^∗∗∗^		51.78^∗∗∗^	

*N = 375; *^∗∗^p* < 0.01, ^∗∗∗^p < 0.001.*

### Testing for the Moderated Mediation

To test Hypothesis 2, we conducted moderated mediation analyses with three regression models as outlined by Muller et al. (2005). In the first model, the moderating effect of impulsivity on the way in which sensation seeking impact on online gaming addiction was estimated. In the second model, the moderating effect of impulsivity on the way in which sensation seeking impact on affective associations with online games was estimated. In the third model, the moderating effect of impulsivity on both the partial effect of affective associations on online gaming addiction and the residual effect of sensation seeking on online gaming addiction were estimated. All variables were standardized to reduce multicollinearity.

**FIGURE 1:**
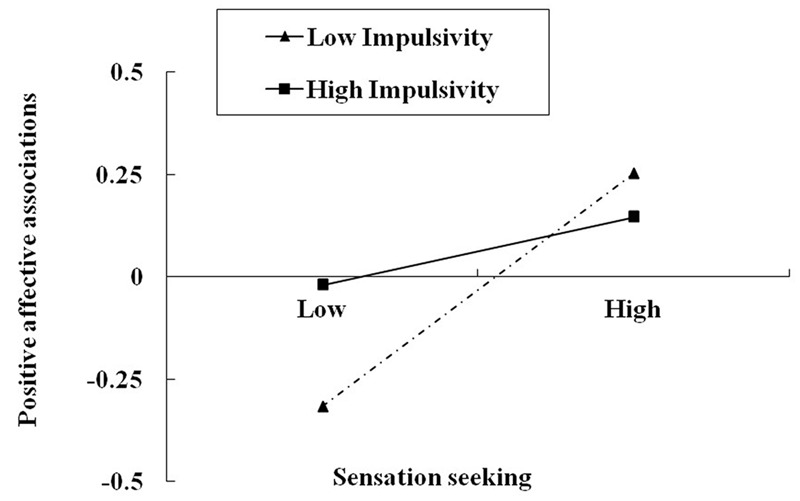
**Positive affective associations with online games as a function of sensation seeking and impulsivity.** Low and high refer to values 1 standard deviation below and above the mean, respectively.

**FIGURE 2:**
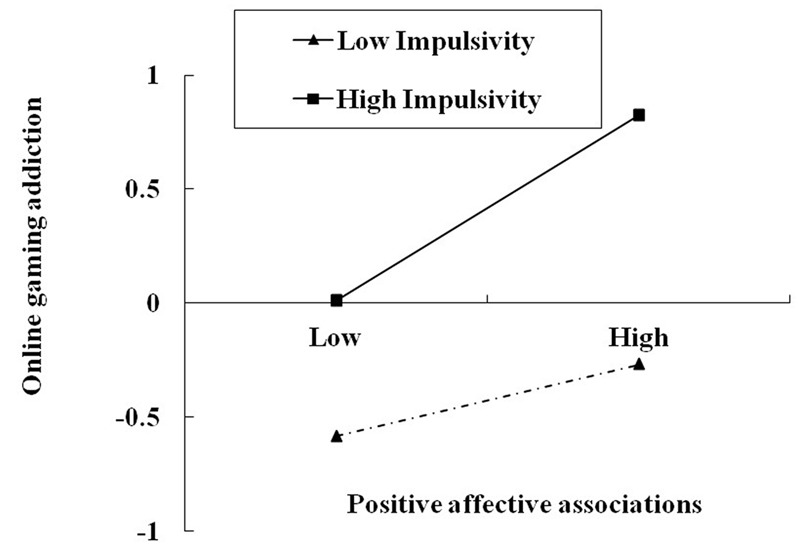
**Online gaming addiction in adolescence as a function of positive affective associations and impulsivity.** Low and high refer to values 1 standard deviation below and above the mean, respectively.

In the first model (**Table 3**), an overall effect of sensation seeking on online gaming addiction was found, *b* = 0.105, *p* < 0.05. This effect was not moderated by impulsivity, *b* = -0.057, *p* > 0.05. In the second model, the mediator, affective associations, was the criterion. There was a main effect of sensation seeking, *b* = 0.184, *p* < 0.001, and a significant sensation seeking × impulsivity interaction effect on affective associations, *b* = -0.105, *p* < 0.05. To facilitate the interpretation of this interaction, we plotted how sensation seeking was related to affective associations at low and high levels of impulsivity (i.e., at 1 SD below and above the mean, respectively, **Figure 1**). Simple slope testing revealed that for low impulsivity adolescents, higher sensation seeking was associated with higher positive affective associations, *b* = 0.285, *p* < 0.001. However, for adolescents with high impulsivity, the effect of sensation seeking on affective associations was non-significant, *b* = 0.084, *p* = 0.249. Finally, the third model showed that the effect of affective associations on online gaming addiction was significant, *b* = 0.422, *p* < 0.001, and this effect was moderated by impulsivity, with a significant affective associations × impulsivity interaction, *b* = 0.125, *p* < 0.01. We also plotted the predicted online gaming addiction against affective associations at low and high levels of impulsivity (**Figure 2**). Simple slope testing showed that for high impulsivity adolescents, affective associations were associated with online gaming addiction, *b* = 0.532, *p* < 0.001. For low impulsivity adolescents, the effect of affective associations on online gaming addiction was weaker, *b* = 0.334, *p* < 0.001.

**Table 3:** Testing the moderated mediation effects of sensation seeking on online gaming addiction in adolescence.

	Model 1 (criterion online gaming addiction)		Model 2 (criterion affective associations)		Model 3 (criterion online gaming addiction)	
Predictors	*b*	*t*	*b*	*t*	*b*	*t*
Sensation seeking	0.105	2.120^∗^	0.184	3.605^∗∗∗^	0.038	0.841
Impulsivity	0.290	5.826^∗∗∗^	0.048	0.938	0.282	6.346^∗∗∗^
Sensation seeking × Impulsivity	-0.057	-1.147	-0.105	-2.064^∗^	-0.035	-0.778
Positive affective associations					0.422	9.402^∗∗∗^
Positive affective associations × Impulsivity					0.125	2.787^∗∗^
*R*^2^	0.112		0.054		0.304	
*F*	15.557^∗∗∗^		7.036^∗∗∗^		32.181^∗∗∗^	

*N = 375; ^∗^*p* < 0.05, ^∗∗^*p* < 0.01, ^∗∗∗^*p* < 0.001.*

## Discussion

In the current study, we tested a moderated mediation model in which the effect of sensation seeking on online gaming addiction through positive affective associations was moderated by impulsivity. This study contributes to a growing body of literature in at least three ways.

First, our results offer support for the biosocial-affect pathway to online gaming addiction, such that high sensation seeking was associated with high level of positive affective associations with online games, which subsequently was associated with a high likelihood of online gaming addiction. This pathway is consistent with the biosocial-affect model, which posits that sensation seeking influences problem behavior through the formation of affective association toward problem behaviors (Romer and Hennessy, 2007). Adolescents higher in sensation seeking have more positive affective associations with online games, which in turn contribute to online gaming addiction in adolescence. The influence of positive affective associations on online gaming addiction is congruent with the affect heuristic model (Slovic et al., 2007), and also extends the behavioral affective associations model (Kiviniemi et al., 2007; Kiviniemi and Duangdao, 2009) to online gaming addiction, as these models usually account for health-related behaviors, such as behaviors in physical activities (Kiviniemi et al., 2007), food choice (Walsh and Kiviniemi, 2014), and smoking (Wilkinson, 2010). This key finding provides support for and extends earlier studies which have found that affective factors, such as perceived enjoyment, are associated with excessive use of online games (Lee et al., 2014).

Second, our results offer support for the Dual Systems Model. We found impulsivity moderated the impact of affective associations on online gaming addiction. As impulsivity increases, the association between affective associations and online gaming addiction becomes stronger. This pattern of findings indicates that impulsivity strengthens the relationship between positive affective associations and online gaming addiction. This finding provides direct evidence for the Dual Systems Model. To some extent, vulnerability to online gaming addiction is the product of high positive affective associations with online games and low impulse control. Higher levels of positive affective associations with online games impel adolescents toward online games; at the same time, immature self-control capabilities cannot restrain this impulse (Steinberg et al., 2008; Steinberg, 2010a). That is, the cognitive control systems of highly impulsive adolescents are relatively “weak,” and their online gaming behavior may be more likely to be guided by affective associations with online games. To our knowledge, it is the first study that applies the Dual Systems Model to adolescent online gaming addiction research.

Surprisingly, we found that impulsivity moderated the influence of sensation seeking on affective associations. In particular, as impulsivity decreased, adolescents higher in sensation seeking were more likely to have favorable affect toward online games. Perhaps adolescents low in impulsivity may inhibit the craving for playing online games and reasonably arrange online game time. In this context, it is not difficult to understand that adolescents with high sensation seeking would be more likely to enjoy online games.

Our study has important practical implications. First, our findings can help practitioners understand how sensation seeking is associated with online gaming addiction, providing reliable evidence for target interventions. For instance, lowering positive affective associations with online games may buffer some of the detrimental effect of sensation seeking on online gaming addiction in adolescence. Thus, it may be useful to consider intervention approaches that target these affective associations with online games. Using an implicit priming paradigm to experimentally manipulate participants’ affective associations with fruit, Walsh and Kiviniemi (2014) found that participants in the positive priming condition chose fruit more often compared to the negative priming condition. Future research may apply such techniques (e.g., repeatedly paired online games related images with neutral words or images) to examine whether adolescents’ affective associations with online games can be altered. Second, given that the risky effect of positive affective associations on online gaming addiction is stronger for more impulsive adolescents, manipulating the affective associations toward online games among adolescents with high impulsivity may be more effective for reducing online gaming addiction.

There are several limitations that need to be considered. First, given the cross-sectional nature of this study, we cannot make any causal inferences of the results. Future studies may test the models using longitudinal or experimental designs to achieve a causal conclusion. Second, all variables were collected using self-reported measure, which may result in common method variance problems. Further studies may use multi-method, multi-informant approaches for evaluating variables. Third, our sample included middle adolescents. Middle adolescents are thought to experience a peak of responsiveness to affective cues while still having immature capacities for impulse control (Harden and Tucker-Drob, 2011). The relative strength of the socio-emotional system and cognitive control systems in mid-adolescents differs for early and late adolescents. Thus, our moderation model may not be generalized to early or late adolescents. The current findings also may not be generalized to female adolescents due to the selection of only male adolescents. Fourth, the present study only focuses on the link between positive affective associations, but not negative affective associations, and online gaming addiction. In general, affect can be divided into positive affect and negative affect. Extensive evidence has shown that positive and negative affect are independent of each other (Diener et al., 1985). So are positive and negative affective associations with stimulus (Shu and Peck, 2011). In the study of Hou and Fang (2014), the positive affective associations, not the negative affective associations with online games were found to be related to the length of time that an individual maintained online gaming behavior, suggesting the different roles of positive and negative affective associations with online games in the maintenance of online gaming addiction. Future studies should investigate whether negative affective associations influence online gaming addiction. In addition, future studies need to examine how affective associations toward online games are formed, which remains unclear.

## Conclusion

We reported how sensation seeking and when impulsivity relate to online gaming addiction in adolescence. Positive affective associations with online games mediated the risky effect of sensation seeking on online gaming addiction in adolescence. Moreover, the risk effect of positive affective associations with online games was moderated by impulsivity. These findings add to our understanding of the mediating and moderating factors that act between sensation seeking and online gaming addiction in adolescence. The results also provide further direct empirical evidence for the Dual Systems Model and biosocial-affect model and a new approach to further explore and understand the mechanism of adolescents’ online gaming addiction.

## Ethics Statement

This study was carried out in accordance with the recommendations of Ethics Committee of Institute of Psychology, South China Normal University with written informed consent from all subjects. All subjects gave written informed consent in accordance with the Declaration of Helsinki. The protocol was approved by the South China Normal University Human Investigation Committee.

## Author Contributions

Conceived and designed the research: WZ, JH. Performed the research: JH, SZ, CY, QZ. Analyzed the data: JH, SZ, CY, QZ. Contributed to the writing of the manuscript: JH, SZ, CY, QZ, WZ.

## Conflict of Interest Statement

The authors declare that the research was conducted in the absence of any commercial or financial relationships that could be construed as a potential conflict of interest.
